# Editorial: Understanding patient nutrition and cancer progression

**DOI:** 10.3389/fonc.2024.1518083

**Published:** 2024-11-25

**Authors:** Ciro Isidoro, Ioannis Ntanasis-Stathopoulos

**Affiliations:** ^1^ Department of Health Sciences, Università del Piemonte Orientale, Novara, Italy; ^2^ Department of Clinical Therapeutics, School of Medicine, National and Kapodistrian University of Athens, Athens, Greece

**Keywords:** cancer metabolism, cachexia, dietary therapy, fasting, nutrition support, micronutrients

Cancer growth, with its high demand of metabolites and energy, causes profound alterations in the food balance and the general homeostasis of the patient.

A range of factors, including circulating inflammatory cytokines in response to tumor and chemo- and radiotherapy, negative mood, and changes in taste and smell, severely reduce food intake in cancer patients (1, 2). Additionally, head-neck cancers and gastrointestinal cancers may cause discomfort and pain when consuming food, leading to giving up eating (3). Consequently, malnutrition is highly prevalent in cancer patients (4), and this is known to affect their adherence and response to therapy, prognosis, and quality of life (5).

This prevalence increases in patients under therapy (6), and further contributes to cachexia (7), a condition of progressive body mass wasting which associates with reduced immune response and patient compliance (8).

Nonetheless, for long time malnutrition in cancer patients has been overlooked (9).

Historically, nutritional management of cancer patients has focused exclusively on limiting the chemotherapy-induced unpleasant effects (such as vomiting and diarrhea) by excluding certain foods and attempting to counteract the weight loss associated with cachexia by parenteral administration of hyper-proteic and hyper-lipidic fluids. Typically, nutritional intervention would be part of the palliative care reserved for terminally ill patients (10).

Studies conducted in recent decades have radically changed this approach, and convinced clinicians to consider nutrition an important ally in the management of cancer patients (11). There are now compelling evidence supporting the beneficial effects of fasting and fasting-mimetic diets, caloric (protein and glucose) restriction diets, nutraceuticals with caloric restriction mimetic properties, and supplementation of micronutrients and vitamins in the management of cancer patients, particularly by reducing the unwanted side effects and reinforcing the efficacy of chemo- and radio-therapy (12). Nutritional intervention as neo-adjuvant therapy also helps the patient to better prepare for surgery and recover afterwards (13).

In this Research Topic of Frontiers in Oncology we have collected articles that delve into the relationship between diet, cancer cell metabolism, cancer growth and patient performance and compliance to the treatments.

Bossi et al. discusses the challenges and strategies associated with managing malnutrition in oncology patients, highlighting the necessity of incorporating nutritional support into cancer care. The authors delineate significant controversies, including timing, risk assessment, and types of nutritional interventions, that influence the management of malnutrition. The authors propose a personalized strategy, suggesting early nutrition screenings and interventions customized to the specific needs of patients to enhance outcomes. The paper also discusses future perspectives, particularly the potential for precision nutrition tailored to tumor type and metabolic requirements, with the goal of improving patient quality of life and treatment effectiveness.

Recent studies elucidate significant connections between iron metabolism, cellular processes, and cancer risk, enhancing the understanding of iron’s intricate role in oncology. Zeidan et al. present a thorough review of epidemiological studies examining the association between iron and cancer, highlighting a more pronounced correlation between heme iron derived from animal sources and elevated risk for several cancers including colorectal, liver, and lung. This review indicates that iron status indicators, such as serum ferritin, may function as risk biomarkers, although these associations vary by cancer type. Genetic predisposition and environmental factors, including diet and lifestyle, significantly influence this risk, highlighting the necessity for more detailed research on these interdependencies.

Yang investigates the causal link between fruit consumption and the risk for colorectal cancer through Mendelian randomization, a technique that utilizes genetic data to establish causality. The research indicates a significant correlation between increased fruit consumption and a decreased risk for colorectal cancer, implying that genetic factors promoting fruit intake may contribute to a lower cancer risk. This study reinforces public health guidelines advocating for increased fruit intake as a preventive strategy for colorectal cancer. This highlights the potential of employing genetic tools in nutritional epidemiology to elucidate diet-cancer relationships.

Sun et al. examine the potential causal relationships between pancreatic cancer and peripheral metabolites through a bidirectional Mendelian randomization approach, which evaluates genetic predispositions that affect metabolite levels and the risk for pancreatic cancer. The research reveals substantial correlations, particularly with lipid and amino acid metabolites, indicating their potential involvement in the progression of pancreatic cancer. Conversely, specific genetic markers associated with pancreatic cancer seem to affect metabolic profiles, indicating a complex, bidirectional relationship. This study emphasizes the significance of metabolomic profiling in elucidating the mechanisms of pancreatic cancer, presenting opportunities for the identification of early biomarkers and therapeutic targets.

Zhang et al. examine the relationship between various metabolic obesity phenotypes and hospitalization rates in patients with multiple myeloma utilizing national retrospective data. Findings indicate that “metabolically unhealthy” obesity, defined by obesity accompanied by metabolic dysfunctions such as insulin resistance, correlates with increased hospitalization rates and prolonged hospital stays in these patients. In contrast, “metabolically healthy” obesity did not significantly affect hospitalization burden, indicating that metabolic health may have a greater influence on multiple myeloma outcomes than obesity alone. This study emphasizes the significance of managing metabolic health to decrease hospitalization and enhance care for patients with multiple myeloma.

## Concluding remarks

Nutritional support in cancer patients not only prevents malnutrition and weakening of the immune response, but also improves the outcome of the therapeutic interventions. Assessment of the nutritional state of the cancer patients (Patient Nutritional Index) helps to predict the clinical evolution and is informative for developing the nutritional therapeutic plan. Therefore, the earliest this assessment is performed and the nutritional interventions starts, the earliest and the largest the benefits for the patient (14, 15).
